# The Diverse Neuromuscular Spectrum of VPS13A Disease

**DOI:** 10.1002/acn3.70198

**Published:** 2025-10-01

**Authors:** Anne Buchberger, Evamaria Riedel, Marie Hackenberg, Alexander Mensch, Stefanie Beck‐Woedl, Joohyun Park, Tobias B. Haack, Bernhard Haslinger, Jan Kirschke, Holger Prokisch, Andreas Hermann, Christian Mawrin, Adrian Danek, Benedikt Schoser, Kevin Peikert, Marcus Deschauer, Isabell Cordts

**Affiliations:** ^1^ Department of Neurology University Hospital rechts der Isar, TUM School of Medicine and Health, Technical University of Munich Munich Germany; ^2^ Department of Neuroradiology University Hospital rechts der Isar, TUM School of Medicine and Health, Technical University of Munich Munich Germany; ^3^ Department of Neurology Martin Luther University Halle‐Wittenberg and University Hospital Halle Halle (Saale) Germany; ^4^ Institute of Medical Genetics and Applied Genomics University of Tübingen Tübingen Germany; ^5^ Center for Rare Diseases University of Tübingen Tübingen Germany; ^6^ Institute of Human Genetics, University Hospital rechts der Isar, TUM School of Medicine and Health Technical University of Munich Munich Germany; ^7^ Genomics for Health in Africa (GHA) Africa‐Europe Cluster of Research Excellence (CoRE) Tübingen Germany; ^8^ Translational Neurodegeneration Section “Albrecht Kossel”, Department of Neurology, University Medical Center Rostock University of Rostock Rostock Germany; ^9^ Center for Transdisciplinary Neurosciences Rostock (CTNR) University Medical Center Rostock, University of Rostock Rostock Germany; ^10^ German Center for Neurodegenerative Diseases (Deutsches Zentrum für Neurodegenerative Erkrankungen—DZNE) Rostock Germany; ^11^ United Neuroscience Campus (UNC) Lund – Rostock Rostock Germany; ^12^ Department of Neuropathology University of Magdeburg Magdeburg Germany; ^13^ Department of Neurology LMU University Hospital, LMU Munich Munich Germany; ^14^ Friedrich Baur Institute at the Department of Neurology LMU University Hospital, LMU Munich Munich Germany

## Abstract

**Objective:**

VPS13A disease (chorea‐acanthocytosis) is a rare neurodegenerative disorder caused by biallelic variants in *VPS13A,* typically presenting with hyperkinetic movement disorders, while neuromuscular signs are often mild. The aim of the project was to investigate the frequency and severity of neuromuscular impairment in VPS13A disease.

**Methods:**

We systematically assessed the neuromuscular involvement in six patients with VPS13A disease. Our evaluation included genetic and clinical data, blood tests, electrophysiological studies, muscle MRI, and tissue samples from muscle and nerve.

**Results:**

Age at clinical onset was 14 to 38 years (median: 37.5). Age at onset of paresis was 27 to 29 years (median: 29). Initial symptoms included seizures (5/6), hyperkinesia (2/6), and muscle weakness (1/6). Neuromuscular signs ranged from hyporeflexia (5/6) to progressive muscle wasting (3/6). Nine *VPS13A* variants were detected, including a novel copy‐neutral inversion. Phosphocreatine kinase was elevated in all cases (498–12,420 U/L; median of highest values: 2230 U/L). Nerve conduction studies revealed sensorimotor axonal neuropathy. Electromyography showed chronic neurogenic changes with high amplitudes, polyphasic potentials, and reduced interference patterns (6/6). Muscle MRI displayed fatty atrophy, most prominently in the calves (5/5). Muscle histology indicated neurogenic and myopathic changes. Electron microscopy of mitochondria and respiratory chain analysis showed no specific pathological findings.

**Interpretation:**

Our findings emphasize the underrecognized neuromuscular spectrum in VPS13A disease, ranging from subclinical signs to severe paresis and sometimes preceding the hyperkinesia that gave rise to the historical term of chorea‐acanthocytosis. A comprehensive understanding of the phenotype is crucial for early diagnosis and appropriate management of VPS13A disease.

## 1 Introduction

VPS13A disease, formerly referred to as chorea‐acanthocytosis, is an autosomal recessive disorder due to biallelic variants in VPS13A, which encodes for VPS13A (chorein) [1, 2]. The disease mechanism is loss of function [3]. While the detailed pathomechanism is under investigation, it is known that VPS13 proteins are involved in bulk lipid transfer at membrane contact sites [4], with VPS13A binding the endoplasmic reticulum to both mitochondria and lipid droplets as well as to the plasma membrane [4, 5, 6, 7, 8]. Besides possible impairment of lipid transport and mitochondrial function, there is evidence for regulatory functions of VPS13A in the cytoskeleton and for cell survival via two pathways, the Lyn kinase and the PI3K pathway, that affect membrane protein phosphorylation and cortical actin expression [9]. Affected individuals characteristically, but not necessarily, show red blood cell acanthocytosis in association with neurological dysfunction [3, 10]. Although clinical findings are typically related to degeneration of the basal ganglia and involve both hyper‐ and hypokinetic movement disorders, including generalized chorea and orofaciolingual dystonia [3, 10], phenotypes can vary drastically. Clinical manifestation may also range from behavioral and cognitive changes to epilepsy [3, 9]. Mild neuromuscular involvement, characterized by elevated phosphocreatine kinase (CK) levels and areflexia, is common [3]. In addition, muscle weakness, fasciculations, and atrophy may occur, with lower limb‐girdle muscular weakness observed in seven of nine cases of a recent study [11]. While one earlier case was described as mimicking motor neuron disease [12], there was evidence of myopathy in others [11, 13]. Individuals suffering from the closely related X‐linked McLeod syndrome (XK disease) [10, 11, 14, 15] appear to show myopathy more often [16], although prominent neurogenic changes occur in patients with XK disease as well [15, 17] and may be considered similarly pronounced as in VPS13A disease [17].

Given the sparse and heterogeneous data available so far, there is a critical need for a systematic exploration of the nature and severity of clinical, paraclinical, imaging, and myopathological neuromuscular involvement in VPS13A disease. Here, we report six cases of VPS13A disease presenting a wide range of neuromuscular involvement, including three patients with moderate to severe paresis. Our study highlights the importance of thorough and repeated phenotyping to ensure early diagnosis, optimal disease management, and to enhance understanding of the underlying pathophysiological mechanisms of VPS13A disease.

## 2 Patients and Methods

Six biological males (five German, one Turkish) with genetically confirmed VPS13A disease were recruited from the University Hospitals of Munich (TUM and LMU) and Rostock and systematically assessed for neuromuscular involvement, regardless of clinically manifest neuromuscular symptoms. Two patients were siblings (IDs 3, 4). Genetic and clinical details of one individual (ID 6) and the cardiac manifestation of three patients (IDs 1, 3, 4) are already on record [18, 19]. All patients gave informed written consent to the scientific use of their clinical and paraclinical data (ethics vote: Biobank Munich 9/15 s; Rostock A 2022‐0058). Clinical, paraclinical, and genetic data were collected during the diagnostic process. Chorein (VPS13A) Western blot in red blood cell (RBC) membranes and acanthocyte counts (wet blood smear) were performed as previously described [20, 21]. Muscle magnetic resonance imaging (MRI) scans were performed on all patients using 3T Philips Ingenia MRI scanners. T2 Dixon sequences [22] were generated, producing in‐phase, out‐of‐phase, fat‐only, and water‐only images, along with quantitative images for assessing muscle infiltration in the thighs. The Morrow scale [23] was employed for evaluating edema, while the Mercuri scale [24] was used for grading fatty infiltration. To derive a disease‐characteristic muscle pattern with respect to our patients' progression stages, we added the two scales to create a combined severity score (1: no involvement; 6: severe involvement). Muscle biopsies (IDs 1, 3, 4, 5) and one sural nerve biopsy (ID 1) were taken during the initial work‐up for CK elevation or muscle weakness, and in one case (ID 6) during surgery for hip arthrosis in the context of known VPS13A disease. Available slides had been stained for hematoxylin and eosin (H&E), Trichrome‐Gomori (IDs 1, 3, 4, 6), and lipid staining (Oil Red or Sudan Orange; IDs 1, 3, 6). In one case (ID 5), new sections from frozen material were prepared and stained accordingly. Immunohistochemistry, including adenosine triphosphatase (ATPase) and cytochrome c oxidase/succinate dehydrogenase (COX/SDH) (IDs 1, 3, 4, 6), respiratory chain analysis (IDs 1, 4, 6), and electron microscopy (IDs 1, 6) were examined where available. Cryopreserved muscle samples were used to assess the respiratory chain activities spectrophotometrically as previously published for IDs 1, 4 [25] and 6 [26]. Statistical data analysis and visualization were performed using RStudio version 1.0.153.

## 3 Results

### 3.1 Clinical, Paraclinical, and Genetic Assessment

Clinical, paraclinical, and genetic findings are summarized in Tables 1 and 2. All individuals were biologically male, all but one (ID 2, Turkish) of German heritage. Age at onset ranged from 14 to 38 (median = 23; SD = 8) years. Age at the last examination ranged from 31 to 45 (median = 38; SD = 5) years. First presenting signs were seizures (5/6; IDs 1–5), hyperkinesia or motor restlessness (2/6; IDs 2, 6), and muscle weakness (1/6; ID 1). Age at onset of neuromuscular signs was 27 to 29 (median = 29; SD = 1) years.

**TABLE 1:** Clinical findings.

ID	Age at onset (years)	Age at onset of muscle weakness (years)	Age at last examination (years)	Symptoms of onset	Psychiatric findings	Muscle weakness (years since onset of muscle weakness)	Ambulatory (non‐ambulatory onset age [years]); devices	Muscle atrophy	Signs of peripheral neuropathy	Neuromuscular syndrome
1	29	29	45	Seizures, progressive paresis	Impulse control disorder	Severe: distal UL 1/5, proximal UL and LL 3/5 (16)	No (32); wheelchair	Severe: proximal and distal UL, proximal LL	Areflexia^b^, reduced vibration sense (6/8)^a^	ALS mimic
2	38	n/a	42	Seizures, orofacial hyperkinesia	Impulse control disorder	None	Yes	None	Areflexia, reduced vibration sense (4–5/8)^a^	Subclinical polyneuropathic syndrome
3	23	27	37	Seizures, low physical endurance		Moderate: proximal UL 4+/5 proximal LL 4−/5 distal LL 4+/5 (10)	Yes; foot orthotics wheelchair (part‐time)	Severe: proximal LL Mild: distal LL	Areflexia, reduced vibration sense (5/8)^a^	Limb‐girdle syndrome
4	14	n/a	32	Seizures	Reduced attention span, disinhibition, psychosis	None	Yes	None	Reduced vibration sense (4–5/8)^a^	Subclinical polyneuropathic syndrome
5	18	29	31	Seizures	Memory impairment, disinhibition	Mild: proximal LL 4+/5 distal LL 4+/5 (2)	Yes	Mild: proximal UL	Areflexia	Motor polyneuropathic syndrome
6	25	29	38	Motor restlessness	Reduced attention span, disinhibition	Moderate: proximal UL 4/5 proximal LL 3/5 distal LL 4/5 (9)	No (34); wheelchair	None	Areflexia	Limb‐girdle syndrome

Abbreviations: ALS, amyotrophic lateral sclerosis; LL, lower limbs; UL, upper limbs.

^a^ Measured on 0‐point scale Rydel‐Seiffer tuning fork.

^b^ Could also be present with myopathy.

**TABLE 2:** Paraclinical findings.

ID	Genotype (variant first allele/variant second allele)	Chorein (RBC)	ACA (%)	CK range (U/L)	NfL (pg/mL)	NCS	EMG	Affected muscles in MRI^b^	Muscle biopsy (age at biopsy)	ECG	Echo	Bicaudate index in MRI^c^	EEG
1	c.8190_8191dup^a^/c.8190_8191dup^a^	Absent	41	900–5083	n/a	Axonal sensorimotor	Neurogenic Myopathic	Shoulder, back, thighs, posterior lower legs	Neurogenic Myopathic 40% COX‐deficiency (35)	Left axis deviation	LVEF mildly reduced^d^	1.57	Generalized slowing
2	c.8743G>T/c.5171T>G	Reduced	14	587–1643	n/a	Axonal sensorimotor	Neurogenic	Medial thighs, lower legs	n/a	Normal	n/a	1.52	Normal
3	c.6059del/chr9:g.79925985_79932966inv^a^	Absent	20–40	2490‐12,420	29	Axonal sensory (ulnar and median nerve only)	Neurogenic	Gluteus, thighs	Neurogenic 2%–3% COX‐deficiency (25)	Sinus arrhythmia	Normal^d^	1.64	Signs of drowsiness
4	c.6059del/chr9:g.79925985_79932966inv^a^	Absent	20–40	2538‐2817	16.5	Axonal motor (peroneal nerve only)	Neurogenic	Back, legs, posterior>anterior	Structurally normal Minimal mitochondrial accumulation (15)	Pathological waves in II, aVL	Normal^d^	1.61	Generalized seizure, focal onset
5	c.283+1G>A^a^/c.9078‐2A>G^a^	Absent	18	608–1061	n/a	Axonal sensorimotor	Neurogenic	Anterior thigh, posterior lower legs	Normal (26)	Sinus brady‐cardia, left axis deviation	Normal	1.61	Mild signs of drowsiness
6	c.556‐290_697‐483del/c.990‐272_1161+1379del	Reduced	+	498–1442	33	Axonal sensorimotor	Neurogenic	Lower legs	Neurogenic COX‐deficiency (35)	Normal	Normal	1.57	Normal

Abbreviations: +,  present (no further information available); ACA, acanthocytes in wet blood smear (normal range: < 6.3% [21]); CK, phosphocreatine kinase (normal range: < 174 U/L); COX, cytochrome c oxidase; ECG, electrocardiography; Echo, echocardiography; EEG, electroencephalography; EMG, electromyography; FD, fatty degeneration; LVEF, left ventricular ejection fraction; MRI, magnetic resonance imaging; n/a, not available; NfL, neurofilament levels in serum (normal range: < 12 pg/mL); RBC, red blood cells.

^a^ Novel variant.

^b^ Fatty degeneration and/or edema.

^c^ Normal range: > 1.8.

^d^ Cardiac manifestation previously published [19].

**FIGURE 1:**
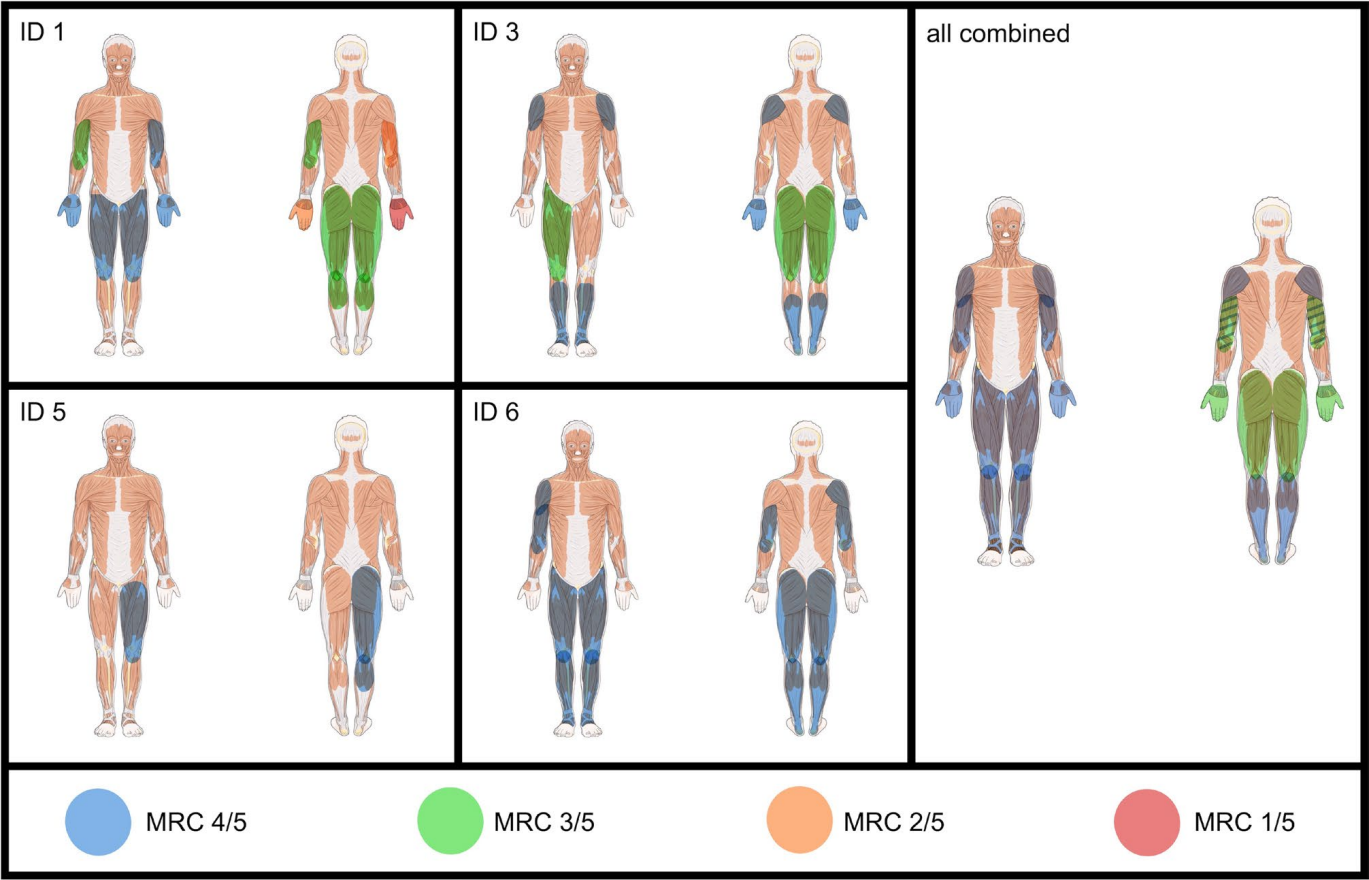
Pattern and severity of paresis in the individual patients (IDs 1, 3, 5, 6; unaffected not shown), and schematic representation of the overall paresis distribution in this VPS13A disease cohort (all combined). Paresis is indicated by color overlay. For the combined pattern, the median MRC score was calculated across all affected muscle groups. MRC = Medical Research Council grading of muscle strength. The artwork used in this figure was adapted from Servier Medical Art (http://https://smart.servier.com/). Servier Medical Art by Servier is licensed under Creative Commons Attribution 4.0. Alterations were color overlay and resizing.

**FIGURE 2:**
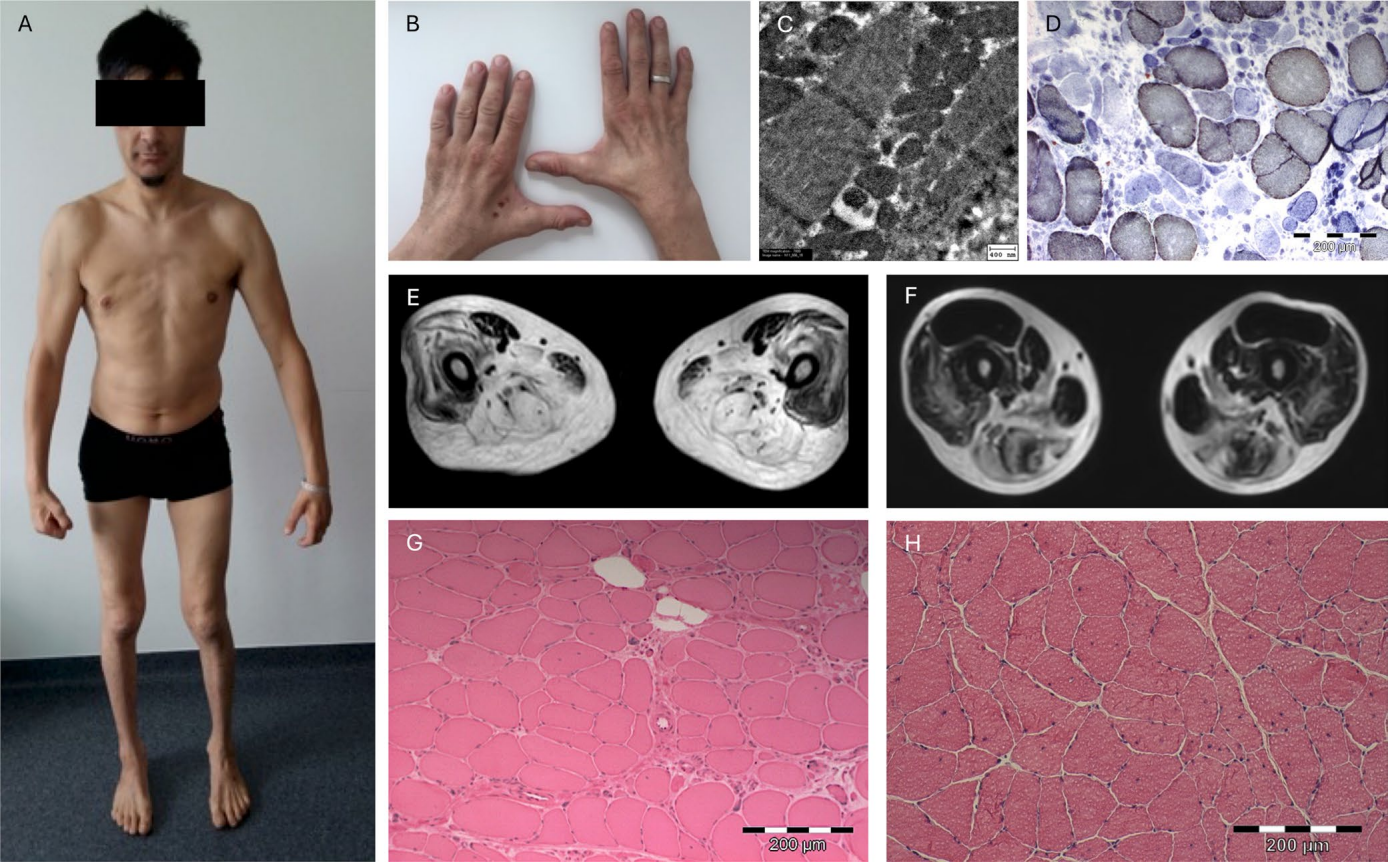
Neuromuscular manifestations of VPS13A disease. Generalized muscle atrophy with severe atrophy of the small hand muscles with split hand sign in one individual (ID 1) (A, B). Muscle MRI of the same patient showed severe fatty degeneration of the posterior thigh muscles (E), similarly seen in another representative patient (ID 3) (F). The muscle biopsy of one patient (ID 1) revealed neurogenic and vacuolar myopathic changes with moderate hypertrophic fibers and mild internalized nuclei in H&E staining (G), multiple COX‐negative fibers in COX/SDH staining (predominantly affecting atrophic fibers, not distributed in a mosaic pattern) (D), and subsarcolemmal accumulated mitochondria of variable sizes, but normal ultrastructure in electron microscopy (C). A muscle biopsy of patient ID 6 showed neurogenic changes with grouping of atrophic and scattered angulated atrophic fibers in H&E staining. Additionally, internalized nuclei were mildly increased (H).

Regarding neuromuscular symptoms, 4/6 reported muscle weakness, while none had sensory complaints. Concerning neuromuscular signs, 4/6 (IDs 1, 3, 5, 6) had mild (1/4; ID 5), moderate (2/4; IDs 3, 6), or severe (1/4; ID 1) muscle weakness, leading to gait impairment in 3/4 (IDs 1, 3, 6; for visualization of pattern and severity of paresis, see Figure 1). Trendelenburg's sign was positive in 2/6 (IDs 3, 6). Gower's sign was observed in half of the cohort (3/6; IDs 1, 3, 6). Split hand sign was present in 1/6 (ID 1) (Figure 2). Hints of the “rubber person gait” were visible in 1/6 (ID 3), but could not be easily differentiated from the paresis. DTR were absent or reduced in 4/6 (IDs 1, 2, 3, 5, 6). Clinical assessment of sensory functions included light touch, vibration, pain, temperature, and proprioception, whereby reduced sense of vibration in 4/6 (IDs 1–4) was the only sign of sensory neuropathy.

The most pronounced sensory involvement was present in cases with mild muscle weakness (IDs 2, 4). In contrast, the patient with the most severe paresis (ID 1) exhibited only a slightly reduced sense of vibration.

Biallelic pathogenic and/or likely pathogenic *VPS13A* variants were detected in 6/6 (Table 2). Six of the nine detected variants were novel. Four cases were sporadic, and two were brothers (IDs 3, 4). Most patients (5/6) carried biallelic null variants (IDs 1, 3, 4, 5, 6). One individual (ID 1) carried a novel duplication in a homozygous state. The two brothers (IDs 3, 4) were compound heterozygotes for a previously described deletion [12, 27, 28], and a novel copy‐neutral inversion spanning exons 36 to 40 that disrupts the open reading frame. The intronic inversion breakpoints were missed by exome sequencing and detected only by genome analysis. In 1/6 (ID 5), two novel splice‐site variants were identified. One individual (ID 6) carried a previously reported deletion of exons 8 and 9 [27], together with a compound heterozygous novel deletion affecting exon 13, which was published upon discovery [18]. In only 1/6 (ID 2), biallelic missense variants were identified. Both variants were novel and classified as likely pathogenic based on multiple lines of evidence, including functional studies showing markedly reduced chorein levels on Western blot, extremely low frequency in control population databases, and computational predictions supporting a deleterious effect (loss of a splice site for variant 1).

Western blot confirmed the diagnosis on the protein level in all patients (6/6) and acanthocytes were significantly increased (6/6; Table 2). CK levels were elevated in 6/6 (498–12,420 U/L; median of highest values: 2230 U/L). Neurofilament light chain (NfL) levels were elevated in 3/3 (16.5–33 pg/mL; median: 29 pg/mL; normal range: < 12 pg/mL; IDs 3, 4, 6).

### 3.2 Electrophysiology

NCS and EMG studies were performed in all patients (6/6, Table 3). Sensory neurography was compatible with neuronopathy or axonal neuropathy in 4/6 (IDs 1, 2, 3, 5, 6), most pronounced in the ulnar nerve. Similarly, motor neurography was compatible with neuronopathy or axonal neuropathy in 4/6 (IDs 1, 2, 4, 5, 6), most frequently in the tibial and peroneal nerves.

**TABLE 3:** Electrophysiological findings.

ID	NCS motor nerves (mV)					NCS sensory nerves (μV)				EMG		
	Median left/right^a^	Ulnar left/right	Radial left/right	Tibial left/right	Peroneal left/right	Median left/right	Ulnar left/right	Radial left/right	Sural left/right	Neurogenic	Myopathic	PSA (intensity)
1	7.6	0.5/No signal	5.3	7.1	Normal	8.9/7.0	6.5/7.3	6.4	Normal	Proximal, distal UL Proximal, distal LL	Proximal, distal UL	Distal UL (+)
2	7.7	Normal	Normal	5.4/3.5	3.8/5.1	6.3	4.1	n/a	Normal	Proximal, distal UL Proximal, distal LL	(Sporadic)	Proximal (+), distal UL (+) Proximal (+), distal LL (++)
3	Normal	Normal	n/a	Normal	Normal	Normal	9.7/11.2	n/a	Normal	Proximal UL^c^ Proximal, distal LL	None	None
4	Normal	Normal	n/a	Normal	4.9	Normal	Normal	n/a	Normal	Proximal, distal UL Proximal, distal LL	None	Distal UL (+) Distal LL (++)
5	6.8^b^	7.3	n/a	8.8	2.3	7.1	9.1	n/a	5	Proximal, distal UL Proximal, distal LL	None	None
6	Normal	Normal	n/a	8.4	5.7/3.8	9.1	4.2	n/a	Normal	Proximal, distal LL^d^	None	None

Abbreviations: +, mild; ++, moderate; EMG = electromyography; NCS = nerve conduction studies; PSA = pathological spontaneous activity, defined as positive sharp waves, fibrillation, or both; normal = above normal cutoff. Cutoff according to laboratory standards (motor nerves: median nerve > 8 mV; ulnar nerve > 8 mV; radial nerve > 8 mV; tibial nerve > 10 mV; peroneal nerve > 6 mV; sensory nerves: median nerve > 10 μV; ulnar nerve > 15 μV; radial nerve > 16 μV; sural nerve > 5.5 μV).

^a^ single values indicate that only one side was measured.

^b^ Distal motor latency = 4.59 ms (normal: < 4.2 ms).

^c^ Distal UL not examined.

^d^ UL not examined.

In EMG, all patients (6/6) showed chronic neurogenic changes with high amplitudes, polyphasic potentials, and reduced interference patterns. In 5/6 (IDs 1–4, 6), this equally affected proximal and distal muscles. Additional myopathic changes with short and small potentials and early recruitment were detected in 1/6 (ID 1). Pathological spontaneous activity (positive sharp waves and/or fibrillations) was present in 3/6 (IDs 1, 2, 4) with equal severity in proximal and distal muscles.

Comparing NCS findings with clinical signs, in cases with moderate to severe sensorimotor neuropathy (IDs 1, 2, 5, 6), 2/4 (IDs 1, 2) presented with reduced vibration sense as the only sensory impairment. Additionally, 2/4 showed no (ID 2) or very mild (ID 5) muscular weakness (MRC 4+/5).

### 3.3 MRI

**FIGURE 3:**
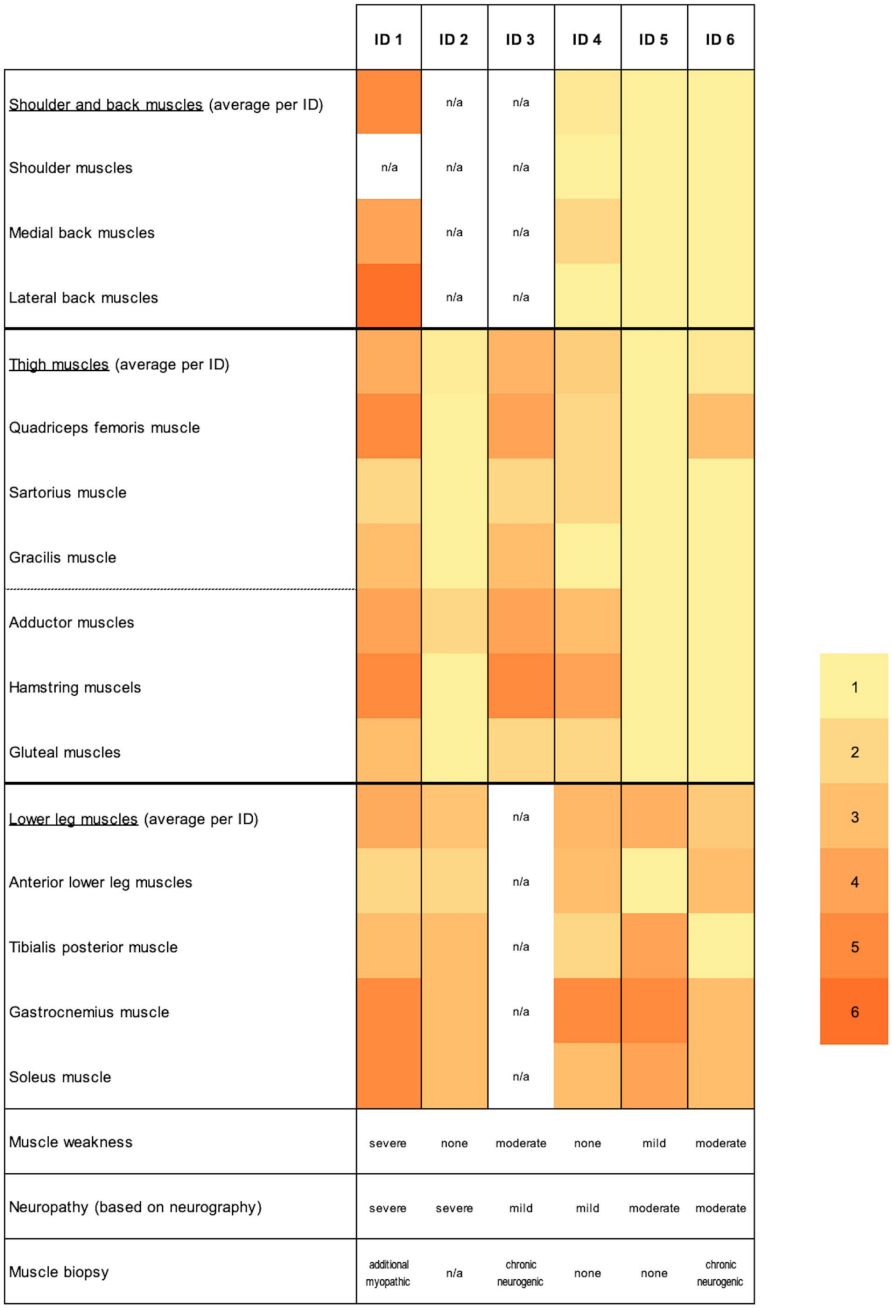
Muscular involvement in MRI according to a combined severity score based on the sum of the Morrow scale [23] for edema and the Mercuri scale [24] for fatty infiltration (1: no involvement; 6: severe involvement). Muscle involvement in MRI is visualized in a heatmap and in relation to other clinical and paraclinical findings. Colors represent the extent of involvement of each muscle by patient. The average extent of muscle involvement by muscle group is shown as a mean colored value. Clinical muscle weakness, neuropathy in NCS, and muscle biopsy results are quantified from normal to severe by patient. MRI = magnetic resonance imaging; n/a = not available; NCS = nerve conduction studies.

MRI scans of the shoulder and/or back muscles were available for 4/6 (IDs 1, 4, 5, 6), thigh muscles for 6/6, and lower leg muscles for 5/6 (IDs 1, 2, 4, 5, 6). To assess muscle involvement patterns, edema (Morrow score 0–2) and fatty degeneration (Mercuri score 1–4) were evaluated and combined into a combined severity score (1–6; Figure 3). The calf muscles were most frequently impaired (5/5; IDs 1, 2, 4, 5, 6) and showed the highest combined severity score (gastrocnemius muscle: 3–5, median 5; soleus muscle: 3–5, median 3; *n* = 5). They also represented the predominant imaging changes in 2/2 patients with mild MRI findings (IDs 2, 6). The gracilis muscle appeared relatively spared (2/6; IDs 1, 3).

Comparing MRI findings with clinical assessments, the prominent involvement of the anterior (4/6; IDs 1, 3, 4, 6) and posterior (3/6; IDs 1, 3, 4) thigh muscles on MRI corresponded with the clinical presentation. In the lower legs, while imaging revealed frequent and severe involvement (5/5), muscular weakness was only mild. In contrast, 6/6 displayed changes in the EMG and NCS of the lower legs, matching the imaging results.

### 3.4 Muscle and Nerve Biopsy

Myopathological analysis was available for 5/6 (IDs 1, 3–6; Table 4; Figure 2). In 3/5 (IDs 1, 3, 6), neurogenic changes were detected, including angulated atrophic fibers (3/3; IDs 1, 3, 6), atrophic fiber grouping (2/3; IDs 1, 3), fiber type grouping (2/3; IDs 3, 6), and fiber type predominance (2/3; IDs 1, 3), of both type 1 (ID 1) and type 2 (ID 3) fibers. Occasionally, muscle fibers in various stages of necrosis (5%–6%) and regenerating fibers were seen (2/5; IDs 1, 3). Additional myopathic features such as internalized nuclei and fiber size variation were present in 2/5 (IDs 1, 6) and 3/5 (IDs 1, 3, 6), respectively. In 2/3 (IDs 3, 6), those findings were accompanying marked chronic neurogenic changes and were therefore most likely secondary to denervation. In 1/6 (ID 1), myopathic changes including fiber size variation (9–125 μm diameter; normal: 40–80 μm) with hypertrophic fibers, internalized nuclei (2%), and endo‐ and perimysial fibrosis with fatty replacement were more prominent. Lipid staining showed multiple fibers with lipid storage in 1/3 (ID 1). COX deficiency was present in 3/5 (IDs 1, 3, 6). Respiratory chain activity was normal in 2/3 (IDs 4, 6) and demonstrated nonspecific reduction of complex I and IV activities in 1/3 (ID 1). Electron microscopy revealed structurally normal mitochondria in 2/2 (IDs 1, 6; Figure 2), some autophagic vacuoles and lipid deposits (1/2; ID 1).

**TABLE 4:** Myopathological findings.

ID	Muscle	Neurogenic						Myopathic			Lipid staining (Oil Red, Sudan Orange)	COX deficiency (%)	Respiratory chain activities	Electron microscopy
		Angulated atrophic fibers	Atrophic fiber grouping	Fiber type grouping	Fiber type predominance	Muscle fiber necrosis	Regenerating fibers	Internalized nuclei (%)	Fiber size variation	Endo−/peri‐mysial fibrosis				
1	Vastus lateralis	+	+	−	+ (type I)	+ (5%)	+	+ (2%)	+	+	Multiple fibers with lipid storage	40	Mildly reduced I and IV activities	Structurally normal mitochondria, autophagic vacuoles, lipid deposits
3	Vastus medialis	+	+	+	+ (type II)	+ (6%) (myo‐phago‐cytes)	+	−	+	−	Normal	2–3	n/a	n/a
4	Thigh	−	−	−	−	−	−	−	−	−	n/a	−	Normal	n/a
5	Gastro‐cnemius	−	−	−	−	−	−	−	−	−	n/a	n/a	n/a	n/a
6	Gluteus medius	+	−	+	−	−	−	+	+	−	Normal	< 10	Normal	Structurally normal mitochondria, few small autophagic vacuoles

Abbreviations: −, absent; +, present; ++, markedly increased; COX, cytochrome c oxidase; n/a, not available.

All individuals with myopathological changes showed clinical muscle weakness, with the most prominent biopsy abnormalities found in the clinically most severely affected case (ID 1). In the two cases with no or mild paresis (IDs 4, 5), no significant myopathological changes were observed.

Histopathological analysis of the sural nerve was available for one individual (ID 1) and showed no significant changes.

## 4 Discussion

Our findings further contribute to understanding the neuromuscular phenotype of VPS13A disease. The spectrum of neuromuscular involvement in our cohort ranged from subclinical changes, which we defined as abnormalities in (para‐)clinical assessments without perceived neuromuscular symptoms, to severe atrophic paresis. Four of six patients showed at least mild paresis, slightly emphasized on the dorsal lower limb girdle, with age at onset of muscle weakness between 27 and 29 years, while two remained only subclinically affected. Muscular weakness in VPS13A disease has been previously reported [3, 11, 12], typically as lower limb‐girdle muscular weakness with onset age ranging from 29 to 53 years [11], with sporadic involvement of distal lower limbs [11, 12]. Isolated reports of severe muscle weakness included one mimicking motor neuron disease [12] and one with initial misdiagnosis of inflammatory myopathy [29], although diagnosis in these cases was not confirmed by Western blot. In our cohort, muscle weakness was one of the most debilitating symptoms in half of the patients. Upper limbs were affected up to split hand sign, which is typically associated with amyotrophic lateral sclerosis [30], proving involvement far beyond the lower limb girdle. Amplitude reduction in sensorimotor ENG was common, reinforcing the previously documented prevalence in literature [3, 11, 31, 32], and highlighting the presence of subclinical neuromuscular changes in patients without neuromuscular symptoms. We found no evidence for reduced nerve conduction velocity, as reported in a small cohort of three [33]. While in earlier studies, EMG changes were solely neurogenic [31], we observed additional myopathic patterns, which have been less recognized [11, 33]. The range of hyperCKemia from ~500 U/L to ~12,400 U/L notably exceeded earlier reports of CK elevations between 500 U/L and 5000 U/L [11], suggesting a broader spectrum of CK changes in VPS13A disease. Muscle MRI consistently demonstrated fatty degeneration or edema, particularly in the calf muscles. While systematic imaging data of VPS13A disease patients is lacking, some heterogeneous involvement of lower leg muscles has been described, with sparing of the soleus muscle reported in one case [34]. As sparing of the soleus muscle was not confirmed in our larger cohort and the anterior and posterior thigh muscles showed regular changes, we were able to revise the pattern.

Molecular genetic testing revealed six novel *VPS13A* variants. These included one frameshift variant, two missense variants, two splice site variants, and a copy‐neutral inversion. Exome sequencing often fails to detect structural variants with intronic breakpoints, as demonstrated in our case. In contrast, genome sequencing offers a more comprehensive view, enabling the detection of complex structural variants. By identifying the first reported *VPS13A* inversion, our findings underscore the superior sensitivity of genome sequencing in detecting disease‐causing structural variants and expand the known mutational spectrum in VPS13A disease. The inversion resulted in a lack of chorein in Western blot analysis in both brothers. However, significant muscle weakness was present in only one of them, indicating no genotype–phenotype correlation in this family. Matching observations were made in siblings with homozygous splice site variants, where only one sibling showed marked muscle weakness [11]. Similar to the homozygous frameshift variant detected in our most severely affected patient, most VPS13A disease‐associated variants are predicted to be loss‐of‐function variants [3] regardless of neuromuscular findings, while missense variants are rare [35]. Thus, no conclusions about a “muscle genotype” can be drawn. In line with this, no clear genotype–phenotype correlation has yet been identified in VPS13A disease [3].

Our cohort of six patients of this rare disease was exclusively male. Given the autosomal recessive mode of inheritance, a more balanced sex distribution would be expected, as observed in other studies.

Regarding the pathomechanism of neurogenic changes, our electrophysiological findings suggest either a neuronopathy or proximally emphasized axonopathy independent of nerve length. Motor nerves were considerably more affected in the lower extremities, while sensory damage was more common in the upper extremities. The sural nerve was notably spared, which has not been reported in VPS13A disease so far. Sural sparing is traditionally associated with immune‐mediated neuropathy [36, 37], with single reports in cases of hereditary neuropathy [38, 39]. One explanation for sural sparing neuropathies is the more proximal measurement site of the sural nerve, reducing sensitivity to distal pathologies [40]. Alternatively, its anatomical protection may limit pressure‐induced damage to the blood‐nerve barrier and subsequent immune‐mediated impairment [41]. Since we observed length‐independent neuropathy without conduction blocks in our cohort, we cannot confirm either theory. In line with our data, sural nerve biopsies showed no significant pathology [28], while in phenotypically [14] and likely pathologically [42] related XK disease, nerve pathology supported axonal sensorimotor neuropathy [17]. The pathophysiological pathway of VPS13A disease likely involves bulk lipid transfer [10, 43]: Since VPS13A has been shown to bind at membrane contact sites between the endoplasmic reticulum and both mitochondria and lipid droplets as well as the plasma membrane (where it forms a complex with XK [7, 8, 44]), biallelic variants in *VPS13A* may lead to impairment of lipid transfer and possibly mitochondrial dysfunction [5, 6, 45, 46]. Pluripotent stem cell‐derived midbrain/hindbrain neurons from VPS13A disease patients showed a distinct mitochondrial trafficking phenotype in proximal axons, suggesting a proximal axonopathy mechanism [47]. As length‐independent axonal impairment may also occur in primary neuronopathy with secondary axonal damage, neuropathological data of spinal neurons are currently lacking to determine the primary site of damage and definitively distinguish between neuronopathy and proximal axonopathy. We confirmed that elevated NfL levels are consistently observed in VPS13A disease, aligning with previous reports of elevated NfL in VPS13A and XK disease [48], therefore supporting the presence of neuro‐axonal damage. Although not specific, this may aid in the diagnostic process, particularly in cases without overt neuromuscular symptoms. However, further studies are needed to explore the potential of NfL as a biomarker for prognosis, correlation with disease course, and phenotypic stratification.

In addition to the evident neurogenic changes, myopathic signs were also present. In half of our cohort, CK levels were highly elevated (above 10‐fold), which is unusual for a neurogenic process. Proximally emphasized muscle weakness with Gower's sign and myopathic EMG changes were indicative of myopathy. One patient's muscle biopsy revealed marked myopathic changes with internalized nuclei and fiber size variation, as well as multiple fibers with lipid storage in Oil Red staining, which has not yet been reported in VPS13A disease but is typical for lipid storage myopathies [49]. In the second most affected individual, subtle myopathic changes accompanied a chronic neurogenic pattern, consistent with secondary myopathic changes after denervation. Lipid staining was normal in this case. Clinical and histopathological changes in VPS13A disease have been previously described as predominantly neurogenic [12, 31, 50]. Nonetheless, some overlapping neuropathic and myopathic muscle biopsy changes have been observed [11, 28, 50, 51, 52, 53, 54]. Myopathic features, such as internalized nucleation and fiber splitting, have been postulated to be secondary to chronic denervation [28, 50, 52, 53]. Other studies interpreted myopathic biopsy patterns as primary [11, 13, 51, 54], for example, due to the detection of nemaline rods in myofibers or the observation of whorled and bizarrely shaped fibers [11, 13, 51]. In our cohort, neurogenic changes were more frequent than myopathic findings, which is compatible with secondary changes in the context of a neurogenic pathology. However, a single patient showed changes that could be indicative of a primary myopathy. The increased lipid storage and marked increase of COX‐deficient fibers in the muscle of this patient with severe atrophic paresis is striking. While lipid accumulation and increase of COX‐negative fibers may suggest bulk lipid transfer and/or mitochondrial dysfunction, our electron microscopy and respiratory chain activities did not provide clear evidence. COX‐deficiency was mainly seen in atrophic fibers without the typical mosaic pattern of mitochondrial myopathy. Therefore, we could not histopathologically confirm a causative mitochondrial or bulk lipid transport disorder in VPS13A disease in muscle. However, we cannot fully exclude the coexistence of a second competing pathology such as lipid storage myopathy or mitochondrial myopathy in this patient, although exome sequencing revealed no indicative variants. In XK disease, similar observations were made: A series of 10 patients found neurogenic changes in all cases, but less prominent myopathic changes in only four cases, which might therefore be classified as secondary to the neurogenic process; however, highly elevated CK levels in some patients and rhabdomyolysis in one patient pointed towards an additional myopathy [17].

## 5 Conclusion

In summary, neuromuscular involvement in VPS13A disease ranges from subclinical changes in NCS, EMG, and MRI to severe atrophic paresis. It is characterized by length‐independent predominant motor neuropathy or neuronopathy, and in severe cases, additional myopathic features may also be observed. Similar to XK disease [17], VPS13A disease frequently leads to muscle weakness. It should therefore not be considered solely a primary movement disorder but a multidimensional condition encompassing a spectrum of neurological signs with varying manifestations, even with muscle weakness as the first and most prominent clinical sign. Thorough phenotyping regarding neuromuscular signs is crucial to support early diagnosis and optimal clinical management of VPS13A disease. Furthermore, understanding the natural history of neuromuscular involvement, which seems to be slowly progressing with frequent clinical manifestation in the late second life decade, is essential for identifying meaningful subgroups and potential biomarkers, ultimately facilitating a comprehensive understanding of the condition and supporting trial readiness.

## Author Contributions

A.B. contributed to the conception of the project, acquisition and interpretation of data, analysis of data, and the drafting, editing, and revision of the manuscript. E.R. contributed to the acquisition of data, analysis and interpretation of data, and the drafting and revision of the manuscript. M.H. contributed to the acquisition of data, analysis and interpretation of data, and the drafting and revision of the manuscript. A.M., S.B.‐W., J.P., T.B.H., B.H., J.K., H.P., A.H., and C.M. contributed to the acquisition of data, and the editing and revision of the manuscript. A.D. contributed to the acquisition of data, interpretation of data, and the editing and revision of the manuscript. B.S. contributed to the acquisition of data, analysis and interpretation of data, and the editing and revision of the manuscript. K.P. contributed to the conception of the project, acquisition of data, interpretation of data, and the editing and revision of the manuscript. M.D. contributed to the conception and supervision of the project, acquisition of data, analysis and interpretation of data, and the editing and revision of the manuscript. I.C. contributed to the conception and supervision of the project, acquisition of data, analysis and interpretation of data, and the drafting, editing, and revision of the manuscript. All authors discussed the results and approved the manuscript.

## Conflicts of Interest

The authors declare no conflicts of interest.

## Data Availability

The data that support the findings of this study are available from the corresponding author upon reasonable request.

## References

[1] L. Rampoldi , C. Dobson‐Stone , J. P. Rubio , et al., “A Conserved Sorting‐Associated Protein Is Mutant in Chorea‐Acanthocytosis,” Nature Genetics 28, no. 2 (2001): 119–120. 11381253 10.1038/88821

[2] S. Ueno , Y. Maruki , M. Nakamura , et al., “The Gene Encoding a Newly Discovered Protein, Chorein, Is Mutated in Chorea‐Acanthocytosis,” Nature Genetics 28, no. 2 (2001): 121–122. 11381254 10.1038/88825

[3] K. Peikert , C. Dobson‐Stone , L. Rampoldi , et al., “VPS13A Disease,” in GeneReviews(®), ed. M. P. Adam , J. Feldman , G. M. Mirzaa , et al. (University of Washington, Seattle, 2002). 20301561

[4] M. Hanna , A. Guillén‐Samander , and P. De Camilli , “RBG Motif Bridge‐Like Lipid Transport Proteins: Structure, Functions, and Open Questions,” Annual Review of Cell and Developmental Biology 16, no. 39 (2023): 409–434. 10.1146/annurev-cellbio-120420-014634 37406299

[5] N. Kumar , M. Leonzino , W. Hancock‐Cerutti , et al., “VPS13A and VPS13C Are Lipid Transport Proteins Differentially Localized at ER Contact Sites,” Journal of Cell Biology 217, no. 10 (2018): 3625–3639. 30093493 10.1083/jcb.201807019 PMC6168267

[6] M. Leonzino , K. M. Reinisch , and P. De Camilli , “Insights Into VPS13 Properties and Function Reveal a New Mechanism of Eukaryotic Lipid Transport,” Biochimica et Biophysica Acta—Molecular and Cell Biology of Lipids 1866, no. 10 (2021): 159003. 34216812 10.1016/j.bbalip.2021.159003 PMC8325632

[7] A. Guillén‐Samander , Y. Wu , S. S. Pineda , et al., “A Partnership Between the Lipid Scramblase XK and the Lipid Transfer Protein VPS13A at the Plasma Membrane,” Proceedings of the National Academy of Sciences of the United States of America 119, no. 35 (2022): e2205425119. 35994651 10.1073/pnas.2205425119 PMC9436381

[8] J. S. Park , Y. Hu , N. M. Hollingsworth , G. Miltenberger‐Miltenyi , and A. M. Neiman , “Interaction Between VPS13A and the XK Scramblase Is Important for VPS13A Function in Humans,” Journal of Cell Science 135, no. 17 (2022): jcs260227. 35950506 10.1242/jcs.260227 PMC9482346

[9] K. Peikert , A. Danek , and A. Hermann , “Current State of Knowledge in Chorea‐Acanthocytosis as Core Neuroacanthocytosis Syndrome,” European Journal of Medical Genetics 61, no. 11 (2018): 699–705. 29253590 10.1016/j.ejmg.2017.12.007

[10] R. H. Walker , K. Peikert , H. H. Jung , A. Hermann , and A. Danek , “Neuroacanthocytosis Syndromes: The Clinical Perspective,” Contact 6 (2023): 25152564231210339. 38090146 10.1177/25152564231210339 PMC10714877

[11] A. Vaisfeld , G. Bruno , M. Petracca , et al., “Neuroacanthocytosis Syndromes in an Italian Cohort: Clinical Spectrum, High Genetic Variability and Muscle Involvement,” Genes (Basel) 12, no. 3 (2021): 344. 33652783 10.3390/genes12030344 PMC7996727

[12] D. Neutel , G. Miltenberger‐Miltenyi , I. Silva , and M. de Carvalho , “Chorea‐Acanthocytosis Presenting as Motor Neuron Disease,” Muscle & Nerve 45, no. 2 (2012): 293–295. 22246890 10.1002/mus.22269

[13] Q. Zheng , L. Zhu , C. Zhang , and L. Jiao , “Nemaline Rods in a Patient of Chorea‐Acanthocytosis With a Novel Pathogenic Mutation of VPS13A Gene,” Neurology India 69, no. 6 (2021): 1848–1849. 34979707 10.4103/0028-3886.333435

[14] K. Peikert , A. Hermann , and A. Danek , “XK‐Associated McLeod Syndrome: Nonhematological Manifestations and Relation to VPS13A Disease,” Transfusion Medicine and Hemotherapy 49, no. 1 (2022): 4–12. 35221863 10.1159/000521417 PMC8832239

[15] H. H. Jung , A. Danek , R. H. Walker , B. M. Frey , and K. Peikert , “McLeod Neuroacanthocytosis Syndrome,” in GeneReviews(®), ed. M. P. Adam , J. Feldman , G. M. Mirzaa , R. A. Pagon , S. E. Wallace , and A. Amemiya (University of Washington, Seattle, 1993).

[16] A. Danek , H. H. Jung , M. A. Melone , L. Rampoldi , V. Broccoli , and R. H. Walker , “Neuroacanthocytosis: New Developments in a Neglected Group of Dementing Disorders,” Journal of the Neurological Sciences 229 (2005): 171–186. 15760637 10.1016/j.jns.2004.11.024

[17] E. Hewer , A. Danek , B. G. Schoser , et al., “McLeod Myopathy Revisited: More Neurogenic and Less Benign,” Brain 130, no. Pt 12 (2007): 3285–3296. 18055495 10.1093/brain/awm269

[18] D. Spieler , A. Velayos‐Baeza , A. Mühlbäck , et al., “Identification of Two Compound Heterozygous VPS13A Large Deletions in Chorea‐Acanthocytosis Only by Protein and Quantitative DNA Analysis,” Molecular Genetics & Genomic Medicine 8, no. 9 (2020): e1179. 32056394 10.1002/mgg3.1179 PMC7507471

[19] S. Quick , F. M. Heidrich , M.‐V. Winkler , et al., “Cardiac Manifestation Is Evident in Chorea‐Acanthocytosis but Different From McLeod Syndrome,” Parkinsonism & Related Disorders 88 (2021): 90–95. 34153885 10.1016/j.parkreldis.2021.05.015

[20] C. Dobson‐Stone , A. Velayos‐Baeza , L. A. Filippone , et al., “Chorein Detection for the Diagnosis of Chorea‐Acanthocytosis,” Annals of Neurology 56, no. 2 (2004): 299–302. 15293285 10.1002/ana.20200

[21] A. Storch , M. Kornhass , and J. Schwarz , “Testing for Acanthocytosis A Prospective Reader‐Blinded Study in Movement Disorder Patients,” Journal of Neurology 252, no. 1 (2005): 84–90. 15654559 10.1007/s00415-005-0616-3

[22] S. Schlaeger , E. Klupp , D. Weidlich , et al., “T2‐Weighted Dixon Turbo Spin Echo for Accelerated Simultaneous Grading of Whole‐Body Skeletal Muscle Fat Infiltration and Edema in Patients With Neuromuscular Diseases,” Journal of Computer Assisted Tomography 42, no. 4 (2018): 574–579. 29613984 10.1097/RCT.0000000000000723

[23] J. M. Morrow , E. Matthews , D. L. R. Rayan , et al., “Muscle MRI Reveals Distinct Abnormalities in Genetically Proven Non‐Dystrophic Myotonias,” Neuromuscular Disorders 23, no. 8 (2013): 637–646. 23810313 10.1016/j.nmd.2013.05.001 PMC3744809

[24] E. Mercuri , A. Pichiecchio , J. Allsop , S. Messina , M. Pane , and F. Muntoni , “Muscle MRI in Inherited Neuromuscular Disorders: Past, Present, and Future,” Journal of Magnetic Resonance Imaging 25, no. 2 (2007): 433–440. 17260395 10.1002/jmri.20804

[25] F. N. Gellerich , M. Deschauer , Y. Chen , T. Müller , S. Neudecker , and S. Zierz , “Mitochondrial Respiratory Rates and Activities of Respiratory Chain Complexes Correlate Linearly With Heteroplasmy of Deleted mtDNA Without Threshold and Independently of Deletion Size. Biochimica et Biophysica Acta (BBA),” Bioenergetics 1556, no. 1 (2002): 41–52. 10.1016/s0005-2728(02)00305-5 12351217

[26] J. C. Fischer , W. Ruitenbeek , F. J. Gabreëls , et al., “A Mitochondrial Encephalomyopathy: The First Case With an Established Defect at the Level of Coenzyme Q,” European Journal of Pediatrics 144, no. 5 (1986): 441–444. 3956532 10.1007/BF00441735

[27] C. Dobson‐Stone and C. Dobson‐Stone , Molecular Genetics of Chorea‐Acanthocytosis (University of Oxford, 2004).

[28] J. Liu , T. Arzberger , A. Radunovic , et al., “1.242 Neuromuscular Findings in Chorea‐Acanthocytosis,” Parkinsonism & Related Disorders 18 (2012): S57.

[29] J. H. Park , J. D. Eun , S. W. Kim , and D. H. Sung , “Neuroacanthocytosis Syndrome Misdiagnosed as Inflammatory Myopathy: A Case Report,” Journal of Electrodiagnosis and Neuromuscular Diseases 23, no. 2 (2021): 67–70.

[30] P. Corcia , P. Bede , P.‐F. Pradat , P. Couratier , S. Vucic , and M. de Carvalho , “Split‐Hand and Split‐Limb Phenomena in Amyotrophic Lateral Sclerosis: Pathophysiology, Electrophysiology and Clinical Manifestations,” Journal of Neurology, Neurosurgery & Psychiatry 92, no. 10 (2021): 1126. 34285065 10.1136/jnnp-2021-326266

[31] L. Rampoldi , A. Danek , and A. P. Monaco , “Clinical Features and Molecular Bases of Neuroacanthocytosis,” Journal of Molecular Medicine (Berlin, Germany) 80, no. 8 (2002): 475–491. 12185448 10.1007/s00109-002-0349-z

[32] H. H. Jung , A. Danek , and R. H. Walker , “Neuroacanthocytosis Syndromes,” Orphanet Journal of Rare Diseases 6 (2011): 68. 22027213 10.1186/1750-1172-6-68 PMC3212896

[33] S. Saiki , K. Sakai , K. Y. Murata , et al., “Primary Skeletal Muscle Involvement in Chorea‐Acanthocytosis,” Movement Disorders 22, no. 6 (2007): 848–852. 17345646 10.1002/mds.21437

[34] S. Ishikawa , N. Tachibana , K. I. Tabata , et al., “Muscle CT Scan Findings in McLeod Syndrome and Chorea‐Acanthocytosis,” Muscle & Nerve 23, no. 7 (2000): 1113–1116. 10883007 10.1002/1097-4598(200007)23:7<1113::aid-mus15>3.0.co;2-6

[35] C. Dobson‐Stone , A. Danek , L. Rampoldi , et al., “Mutational Spectrum of the CHAC Gene in Patients With Chorea‐Acanthocytosis,” European Journal of Human Genetics 10, no. 11 (2002): 773–781. 12404112 10.1038/sj.ejhg.5200866

[36] V. F. d. S. Castro , R. T. G. d. Oliveira , J. D. L. d. Santos , et al., “The Sural‐Sparing Pattern in Clinical Variants and Electrophysiological Subtypes of Guillain‐Barré Syndrome,” Arquivos de Neuro‐Psiquiatria 82 (2024): 1–7. 10.1055/s-0044-1785692 PMC11031252 38641340

[37] N. Pasutharnchat , V. Ratanasirisawad , M. Santananukarn , C. Taychargumpoo , J. Amornvit , and C. Chunharas , “Sural‐Sparing Pattern: A Study Against Electrodiagnostic Subtypes of Guillain–Barre Syndrome,” Clinical Neurophysiology Practice 7 (2022): 266–272. 36248727 10.1016/j.cnp.2022.09.001 PMC9557237

[38] M. Mercan , V. Yayla , S. Altinay , and S. Seyhan , “Peripheral Neuropathy in Tangier Disease: A Literature Review and Assessment,” Journal of the Peripheral Nervous System 23, no. 2 (2018): 88–98. 29582519 10.1111/jns.12265

[39] İ. Koç , G. Koç , B. Özenç , and Z. Odabaşı , “Hereditary Neuropathy With Liability to Pressure Palsy Presenting as Bilateral Foot Drop,” Eurasian Journal of Medicine 55, no. 1 (2023): 90–92. 36861874 10.5152/eurasianjmed.2023.21154 PMC10081114

[40] M. B. Bromberg and J. W. Albers , “Patterns of Sensory Nerve Conduction Abnormalities in Demyelinating and Axonal Peripheral Nerve Disorders,” Muscle & Nerve 16, no. 3 (1993): 262–266. 8383290 10.1002/mus.880160304

[41] T. Umapathi , Z. Li , K. Verma , and N. Yuki , “Sural‐Sparing Is Seen in Axonal as Well as Demyelinating Forms of Guillain–Barré Syndrome,” Clinical Neurophysiology 126, no. 12 (2015): 2376–2380. 25743269 10.1016/j.clinph.2015.01.016

[42] J. S. Park and A. M. Neiman , “XK Is a Partner for VPS13A: A Molecular Link Between Chorea‐Acanthocytosis and McLeod Syndrome,” Molecular Biology of the Cell 31, no. 22 (2020): 2425–2436. 32845802 10.1091/mbc.E19-08-0439-T PMC7851852

[43] T. J. Melia and K. M. Reinisch , “A Possible Role for VPS13‐Family Proteins in Bulk Lipid Transfer, Membrane Expansion and Organelle Biogenesis,” Journal of Cell Science 135, no. 5 (2022): jcs259357. 35267021 10.1242/jcs.259357 PMC8976877

[44] Y. Ryoden , K. Segawa , and S. Nagata , “Requirement of Xk and VPS13A for the P2X7‐Mediated Phospholipid Scrambling and Cell Lysis in Mouse T Cells,” Proceedings of the National Academy of Sciences of the United States of America 119, no. 7 (2022): e2119286119. 35140185 10.1073/pnas.2119286119 PMC8851519

[45] W. M. Yeshaw , M. van der Zwaag , F. Pinto , et al., “Human VPS13A Is Associated With Multiple Organelles and Influences Mitochondrial Morphology and Lipid Droplet Motility,” eLife 8 (2019): e43561. 30741634 10.7554/eLife.43561 PMC6389287

[46] B. D. M. Bean , S. K. Dziurdzik , K. L. Kolehmainen , et al., “Competitive Organelle‐Specific Adaptors Recruit Vps13 to Membrane Contact Sites,” Journal of Cell Biology 217, no. 10 (2018): 3593–3607. 30018089 10.1083/jcb.201804111 PMC6168272

[47] H. Glaß , P. Neumann , A. Pal , et al., “Combined Dendritic and Axonal Deterioration Are Responsible for Motoneuronopathy in Patient‐Derived Neuronal Cell Models of Chorea‐Acanthocytosis,” International Journal of Molecular Sciences 21, no. 5 (2020): 1797. 32151030 10.3390/ijms21051797 PMC7084777

[48] K. Peikert , K. Akgün , C. Beste , et al., “Neurofilament Light Chain in Serum Is Significantly Increased in Chorea‐Acanthocytosis,” Parkinsonism & Related Disorders 80 (2020): 28–31. 32932025 10.1016/j.parkreldis.2020.09.004

[49] E. M. Pennisi , M. Garibaldi , and G. Antonini , “Lipid Myopathies,” Journal of Clinical Medicine 7, no. 12 (2018): 472. 30477112 10.3390/jcm7120472 PMC6306737

[50] L. C. Limos , A. Ohnishi , T. Sakai , N. Fujii , I. Goto , and Y. Kuroiwa , ““Myopathic” Changes in Chorea‐Acanthocytosis. Clinical and Histopathological Studies,” Journal of the Neurological Sciences 55, no. 1 (1982): 49–58. 6213738 10.1016/0022-510x(82)90169-1

[51] Y. Tamura , K. Matsui , H. Yaguchi , M. Hashimoto , and K. Inoue , “Nemaline Rods in Chorea‐Acanthocytosis,” Muscle & Nerve 31, no. 4 (2005): 516–519. 15660376 10.1002/mus.20243

[52] A. Ohnishi , Y. Sato , H. Nagara , et al., “Neurogenic Muscular Atrophy and Low Density of Large Myelinated Fibres of Sural Nerve in Chorea‐Acanthocytosis,” Journal of Neurology, Neurosurgery, and Psychiatry 44, no. 7 (1981): 645–648. 7288454 10.1136/jnnp.44.7.645 PMC491072

[53] M. E. Alonso , F. Teixeira , G. Jimenez , and A. Escobar , “Chorea‐Acanthocytosis: Report of a Family and Neuropathological Study of Two Cases,” Canadian Journal of Neurological Sciences 16, no. 4 (1989): 426–431. 10.1017/s0317167100029516 2804805

[54] Y. Kageyama , K. Matsumoto , K. Ichikawa , et al., “A New Phenotype of Chorea‐Acanthocytosis With Dilated Cardiomyopathy and Myopathy,” Movement Disorders 22, no. 11 (2007): 1669–1670. 17516458 10.1002/mds.21556

